# Molecular characterization of colorectal adenoma and colorectal cancer via integrated genomic transcriptomic analysis

**DOI:** 10.3389/fonc.2023.1067849

**Published:** 2023-07-21

**Authors:** Peng Pan, Jingnan Li, Bo Wang, Xiaoyan Tan, Hekun Yin, Yingmin Han, Haobin Wang, Xiaoli Shi, Xiaoshuang Li, Cuinan Xie, Longfei Chen, Lanyou Chen, Yu Bai, Zhaoshen Li, Geng Tian

**Affiliations:** ^1^ Department of Gastroenterology, Shanghai Changhai Hospital, Shanghai, China; ^2^ Department of Gastroenterology, Peking Union Medical College Hospital, Beijing, China; ^3^ Department of Science, Geneis Beijing Co., Ltd., Beijing, China; ^4^ Department of Gastroenterology, Maoming People's Hospital, Maoming, China; ^5^ Department of Gastroenterology, Jiangmen Central Hospital, Jiangmen, China; ^6^ Department of Bioinformatics, Boke Biotech Co., Ltd., Wuxi, China

**Keywords:** colorectal adenoma, colorectal cancer, multi-omics, random forest, monitor, screening

## Abstract

**Introduction:**

Colorectal adenoma can develop into colorectal cancer. Determining the risk of tumorigenesis in colorectal adenoma would be critical for avoiding the development of colorectal cancer; however, genomic features that could help predict the risk of tumorigenesis remain uncertain.

**Methods:**

In this work, DNA and RNA parallel capture sequencing data covering 519 genes from colorectal adenoma and colorectal cancer samples were collected. The somatic mutation profiles were obtained from DNA sequencing data, and the expression profiles were obtained from RNA sequencing data.

**Results:**

Despite some similarities between the adenoma samples and the cancer samples, different mutation frequencies, co-occurrences, and mutually exclusive patterns were detected in the mutation profiles of patients with colorectal adenoma and colorectal cancer. Differentially expressed genes were also detected between the two patient groups using RNA sequencing. Finally, two random forest classification models were built, one based on mutation profiles and one based on expression profiles. The models distinguished adenoma and cancer samples with accuracy levels of 81.48% and 100.00%, respectively, showing the potential of the 519-gene panel for monitoring adenoma patients in clinical practice.

**Conclusion:**

This study revealed molecular characteristics and correlations between colorectal adenoma and colorectal cancer, and it demonstrated that the 519-gene panel may be used for early monitoring of the progression of colorectal adenoma to cancer.

## 1 Introduction

Although the mortality rate of colorectal cancer is declining, it remains one of the top three causes of cancer-related deaths (1–7). The occurrence of cancer is a multi-step process, and most malignant colorectal cancers are caused by pre-existing benign tumors (8, 9). Colorectal cancer is caused by the activation of oncogene mutations and the inactivation of tumor suppressor genes, the latter of which is the main cause (10–14). The specific process begins with the abnormal growth of colonic epithelium, which transforms into colorectal adenoma and finally into cancer (15–17). Chemotherapy and radiation therapy are the main modes of treatment for tumors, but these treatments are quite taxing for patients and the results are not always ideal (18–22). Therefore, the detection and treatment of cancer at an early stage of development is critical. Preventive intervention during this period can preclude the development of cancer and potentially reverse the process of cancer development (23).

Colonoscopy plays an important role in the diagnosis and treatment of colorectal cancer during its formation and development (24). However, the disadvantages of colonoscopy include patients’ reluctance to undergo the procedure, possible complications, and economic burden for patients (23). Colorectal adenoma generally refers to a raised lesion that protrudes from the rectal mucosa surface into the intestinal cavity. Colonoscopy can detect polyps and aid doctors in polyp removal, but it cannot identify whether a polyp is neoplastic (15). Gong et al. established a real-time quality improvement system to monitor the speed of colonoscopy sampling in real time, thereby improving the effectiveness of colonoscopy in detecting adenomas (24). In another study, regular aspirin was shown to reduce the incidence of colorectal adenomas, and other non-steroidal anti-inflammatory drugs (NSAIDs) were shown to mitigate the risk of colorectal tumors (25). Moreover, obesity has been reported as a poor prognostic factor, and weight control has been shown to decrease the risk of colorectal adenoma progression (26, 27). Therefore, establishing an approach to discerning the prognosis of colorectal adenoma can increase the early screening detection of colorectal cancers and avoid unnecessary treatment for benign colorectal adenoma.

Numerous studies have explored the relationship between colorectal adenoma and colorectal cancer. By comparing the somatic mutation research of colorectal adenoma and colorectal cancer, a supervised random deep forest model was established in (23). In (28), whole-exome sequencing and targeted sequencing methods were used to describe the somatic mutations of colorectal precancerous lesions, and then through comparison with colorectal cancer, a colorectal adenoma genome map was established to identify the direction of colorectal adenoma molecular markers of cancer development. The map indicated that, during the process of colorectal adenoma development into colorectal cancer, genome stability decreased and mutations accumulated, resulting in alterations in RNA expression. The Consensus Molecular Subtype (CMS) is a colorectal cancer classification system based on RNA expression. In (15), it was found that colorectal adenomas can be classified into CMS types. Further study revealed that the distribution of CMS types in colorectal adenomas was consistent with the proportion of adenomas that progressed to colorectal cancer. Colorectal adenomas are also called colorectal neoplastic polyps, which belong to a category of polyps. Polyps can be divided into cancer adjacent polyps (CAP) and cancer-free polyps (CFP). The characteristics of CAP and CFP tissues based on their genes, expression, and methylation patterns are used to define molecules related to the progression of polyps to cancer, and to provide candidate markers for screening. Adenoma and normal mucosa transcripts from the same individual were explored in (29), and it was found that a key feature of the transformation process was the remodeling of the Wnt pathway. KIAA1199 is a new target of the Wnt signaling pathway and a potential indicator of colorectal adenoma transformation. In (30, 31), an unidentified gene locus (chR16: hCG_1815491) named colorectal neoplasia differentially expressed (CRNDE) was found to be activated in the early stage of colorectal cancer; further research found that the single CRNDE transcript can be used as a tissue and plasma biomarker of colorectal adenoma and cancer with high sensitivity and specificity. The human large intestine has many complex bacterial communities. Studying the relationship between these bacterial communities and colorectal adenomas in (32, 33) revealed that changes in the composition of the bacterial communities associated with adenomas may be related to the etiology of colorectal cancer. This provides a new direction for the prevention of colorectal adenoma and colorectal cancer. In terms of technology, the use of next-generation sequencing (NGS) technology to study colorectal adenoma and colorectal cancer-related genes is a superior choice. Studies have shown that a targeted sequencing platform using NGS technology can be used in the clinic to provide comprehensive data on genetic changes (5, 34, 35).

Past research has improved our understanding of the natural history and treatment of colorectal adenomas and uncovered the advantages and disadvantages of general methods of detecting and removing adenomas. Although some studies have investigated colorectal adenoma and colorectal cancer based on the NGS platform, few have utilized multi-omics data. With the advancement of science and technology, improvements in risk stratification, adenoma detection, monitoring intervals, and screening have contributed to the prevention of colorectal cancer (36). The objective of the present study was to develop an NGS panel capable of DNA&RNA Parallel Capture for the exploration of molecular characterization of colorectal adenoma and cancer. Comparing the separate capture of DNA and RNA with co-capture of DNA and RNA, the latter brings about several advantages. Firstly, detections in RNA could complement detections in DNA (37). Though targeted capture at the DNA level can precisely identify genomic variation such as single nucleotide variants, insertions, deletions and breakpoints of structure variation, changes at the DNA level do not necessarily reflect corresponding changes in biological phenotypes. Therefore, detection at the transcriptome or proteome level is essential. According to reports, 12.8% of uncommon fusions identified at the DNA level did not result in abnormal transcription or proteins. In such cases, targeted therapy is not effective in clinical treatment (38). Secondly, co-capturing of DNA and RNA minimizes molecular difference between DNA and RNA caused by tumor heterogeneity (39). Thirdly, co-capture of DNA and RNA is a simpler process that requires only one hybridization capture experiment, but allowed obtaining DNA and RNA sequencing results simultaneously. It reduces reagent and sequencing costs and saves experimental time (40).

First, 519 cancer-related genes in the NGS targeted panel—consisting of tumor oncogenes, tumor suppressor genes, and genes associated with target drugs and chemotherapy drugs—were collected from OncoKB (41), Cosmic (42), TCGA (43), and the literature. Specifically, the panel covered 73 drug target genes, 30 chemotherapy drug-related genes, and 74 hereditary tumor genes. Then the potential molecular mechanism of the progression from colorectal adenoma to colorectal cancer was investigated, and common markers and key driver genes were identified to aid clinical application in more accurately determining the potential for disease progression.

## 2 Results

### 2.1 Overview

**Figure 1:**
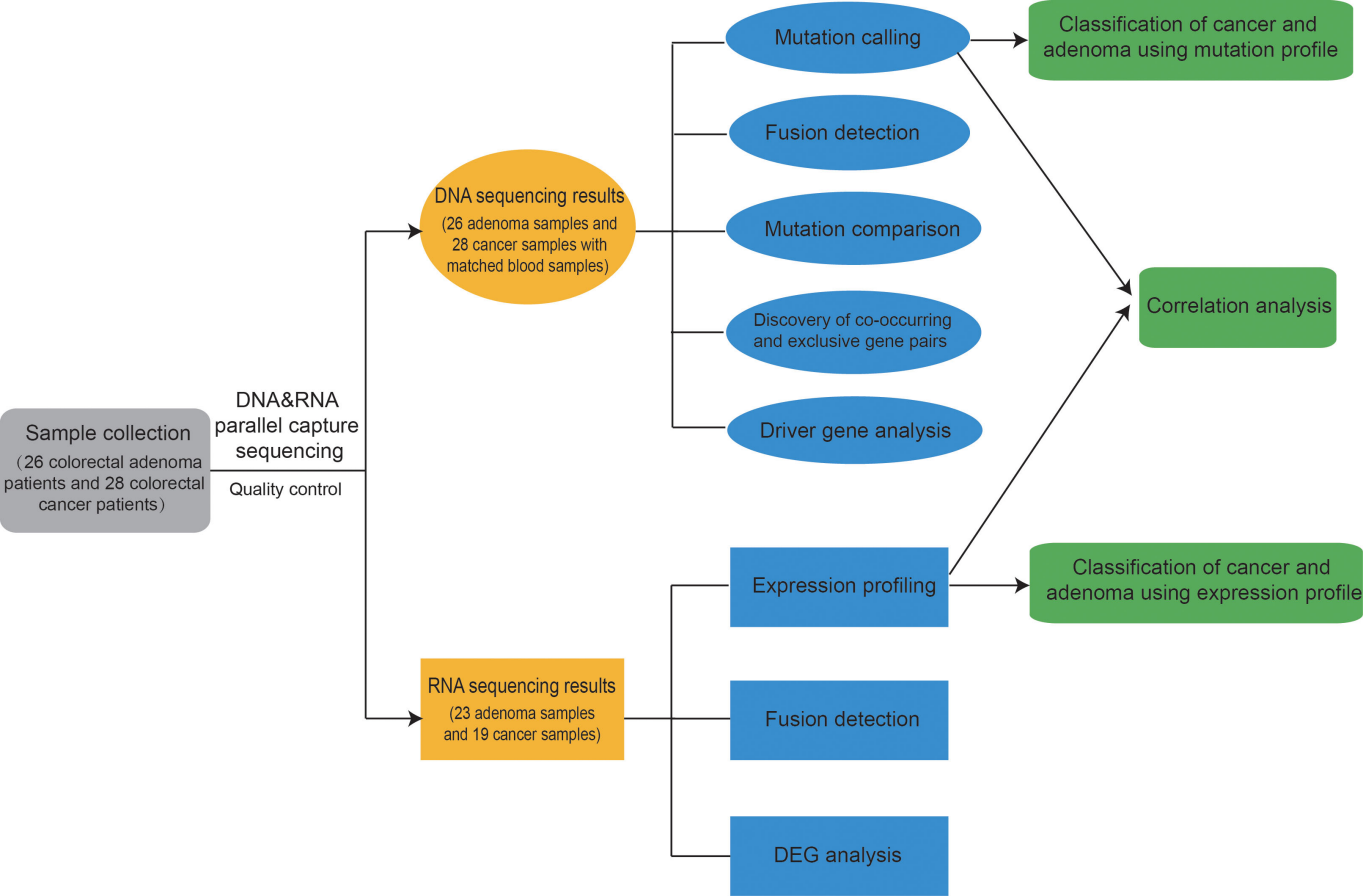
Overview of this study. First, 26 colorectal adenoma patients and 28 colorectal cancer patients were enrolled, and DNA&RNA Parallel Capture was applied on the patients’ adenoma and cancer samples. Mutation profiles and expression profiles were analyzed. Finally, a model was trained for classification between adenoma and cancer samples, achieving good results. Classification results from both expression profiles and mutation profiles could aid in making decisions.

First, 26 colorectal adenoma patients and 28 colorectal cancer patients were enrolled. The clinical information of these patients are listed in **Supplementary Tables 1, 2**. Mutation profiles and expression profiles were obtained for all available patients. Multiple analysis was applied to the mutation profiles and the expression profiles. Finally, a model was trained for classification between adenoma and cancer samples, achieving good results. The entire analysis process is shown in **Figure 1**.

### 2.2 Somatic mutations in the two groups showed similarity in most frequently mutated genes and transitions/transversions ratio

In total, DNA sequencing data of 26 colorectal adenoma and 28 colorectal cancer cases passed the quality control. Thus, somatic mutations were called for those samples using matched blood samples as normal. The mutations are listed in **Supplementary Table 3**.

**Figure 2:**
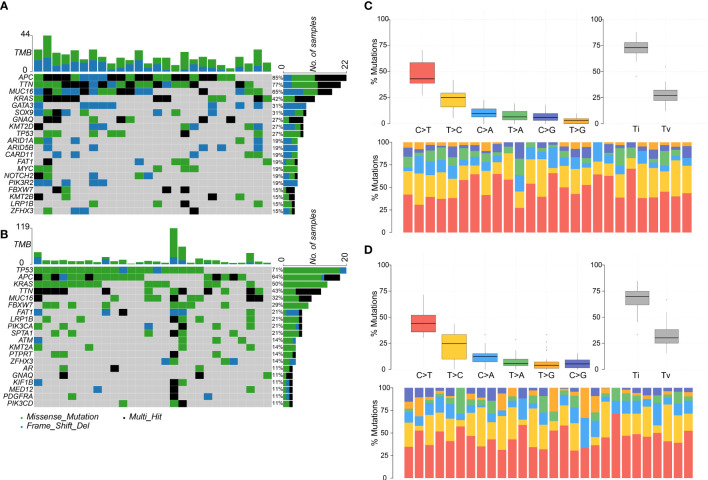
Colorectal adenoma or cancer sample mutation profile analysis. **(A)** Mutation profile summary for 26 adenoma samples. **(B)** Mutation profile summary for 28 cancer samples. **(C)** Transitions and transversions ratio visualization for 26 adenoma samples. **(D)** Transitions and transversions ratio visualization for 28 cancer samples.

The top five most frequently mutated genes in the adenoma group were *APC*, *TTN*, *MUC16*, *KRAS*, and *GATA3*, and the top five most frequently mutated genes in the cancer group were *TP53*, *APC*, *KRAS*, *TTN*, and *MUC16* (**Figures 2A, B**). Hence, the two groups shared four common genes in the top five most frequently mutated genes, namely *APC*, *TTN*, *MUC16*, and *KRAS*. The most frequently mutated gene, *APC*, encodes a tumor suppressor protein that acts as an antagonist of the Wnt signaling pathway. Mutations at specific loci of *APC* and inactivation of *APC* can lead to familial adenomatous polyposis, an autosomal dominant pre-malignant disease that usually progresses to colorectal cancer (44, 45).

Transitions and transversions were also analyzed in the adenoma and cancer groups (**Figures 2C, D**). The two groups tended to harbor more transitions than transversions, had similar Ti/Tv (transition/transversion) ratios, and had C>T as the most frequent mutation type. Interestingly, the top four most frequent mutation types, C>T, T>C, C>A, and T>A, were in the same order in both groups.

Furthermore, the driver genes were analyzed in the two groups. In the adenoma group, *GNAQ*, *KRAS*, *MUC16*, and *TTN* were identified as the driver genes, whereas in the cancer group, only *GNAQ* and *KRAS* were identified as the driver genes. Notably, *GNAQ* and *KRAS* were identified as driver genes in both groups.

### 2.3 TP53, SPTA1, SOX9, and ARID5B were differentially mutated in the two groups

Colorectal cancer patients harbored more mutations in *TP53* and *SPTA1*, two genes that are frequently mutated in breast cancer (46). *TP53* encodes a tumor suppressor protein containing transcriptional activation, DNA binding, and oligomerization domains; this protein responds to diverse cellular stresses to regulate the expressions of the target genes, thereby inducing cell cycle arrest, apoptosis, senescence, DNA repair, or changes in metabolism. *TP53* is closely related to colon cancer (47) and other cancers (48, 49). *SPTA1* encodes molecular scaffold proteins that link the plasma membrane to the actin cytoskeleton. These proteins determine cell shape, arrangement of transmembrane proteins, and organization of organelles. Mutations in *SPTA1* result in a variety of hereditary red blood cell disorders. Furthermore, as reported by Tian et al., *SPTA1* is also related to tumor burden in cholangiocarcinoma (50).

*SOX9* and *ARID5B* were mutated in the adenoma group but not in the cancer group. The protein encoded by *SOX9* acts during chondrocyte differentiation and, with steroidogenic factor 1, regulates transcription of the anti-Müllerian hormone (AMH) gene. *SOX9* has been reported to be positively correlated to tumor size (51). Thus, mutations on *SOX9* might impair the function of the protein encoded by *SOX9*, which might suppress the development of colorectal cancer. *ARID5B* plays a role in the cell growth and differentiation of B-lymphocyte progenitors. It is also reported to be related to the development of acute lymphoblastic leukemia (52). We surmise that *ARID5B* might be a new target for the prevention of colorectal cancer.

### 2.4 Distinct mutually exclusive and co-occurring oncogene patterns were found in the two groups

**Figure 3:**
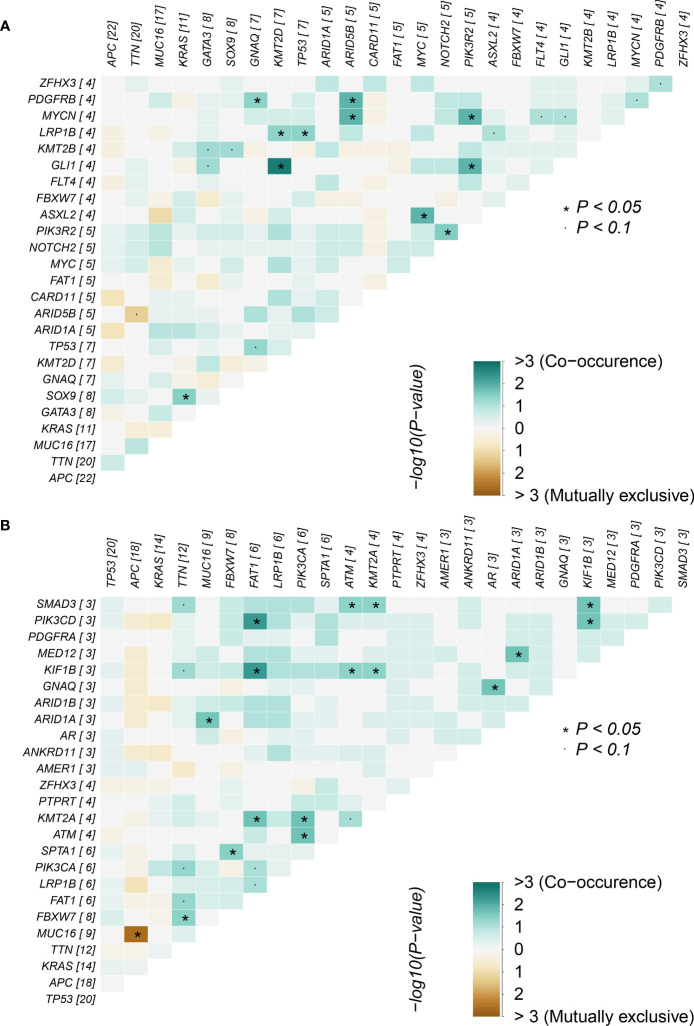
Mutually exclusive and co-occurring gene pairs in **(A)** colorectal adenoma samples and **(B)** colorectal cancer samples.

Correlations between mutations in the adenoma group and cancer group were analyzed (**Figure 3**), focusing on the 10 overlapping mutated genes between the two groups: *APC*, *ARID1A*, *FAT1*, *FBXW7*, *GNAQ*, *KRAS*, *LRP1B*, *MUC16*, *TP53*, *TTN*, and *ZFHX3*. In the adenoma group, co-occurrences related to the 10 genes were found between the gene pairs *GNAQ-PDGFRB*, *KRAS-SOX9*, *LRP1B-KMT2D*, and *LRP1B-TP53*. Co-occurrences gene pair means two genes in the pair co-mutate more often than expected. Similarly, mutually exclusive pair means two genes co-mutate less often than expected. However, no significant mutually exclusive gene pairs were found in the adenoma group. For the same 10 genes, more co-occurrence gene pairs were found: *ARID1A-MUC16*, *ARID1A-MED12*, *FAT1-PIK3CD*, *FAT1-KIF1B*, *FBXW7-TTN*, *FBXW7-SPTA1*, and *GNAQ-AR*. Furthermore, the mutually exclusive gene pair *APC-MUC16* was found in the cancer group. Though correlated gene pairs were found in both groups, no common gene pair appeared in the two groups, indicating that the mutually exclusive and co-occurring oncogene mutations in adenoma patients might help in distinguishing between colorectal adenoma patients and colorectal cancer patients.

### 2.5 Detection of ETV6-NTRK3 fusions in colorectal cancer samples on DNA and RNA levels

We detected three fusions in three colorectal cancer samples on DNA level and one fusion in one colorectal adenoma sample on RNA level (**Table 1**). The fusions detected were all ETV6-NTRK3 fusion. This fusion has been reported in colorectal cancer patients (53–55). The additional fusion was detected only in RNA data, showing the RNA could complement the DNA on the detection of fusion.

**Table 1:** Fusions detected in colorectal cancer samples and adenoma samples.

sample	fusion	Source
15F	ETV6-NTRK3	RNA
519S1	ETV6-NTRK3	DNA
519S3	ETV6-NTRK3	DNA
519S9	ETV6-NTRK3	DNA

### 2.6 151 differentially expressed genes were found between cancer and adenoma samples

As the RNA of some samples did not meet requirements, RNA sequencing data from 19 colorectal cancer samples and 23 adenoma samples passed the quality control, and the expression profiles are listed in **Supplementary Table 4**. We plotted the principal coordinate analysis (PCoA) for the 19 colorectal cancer samples and TCGA samples after batch correction in **Supplementary Figure 1** as the quality control procedure. We found no significant difference between the expression profiles of the selected cancer cohorts and those from TCGA.

**Figure 4:**
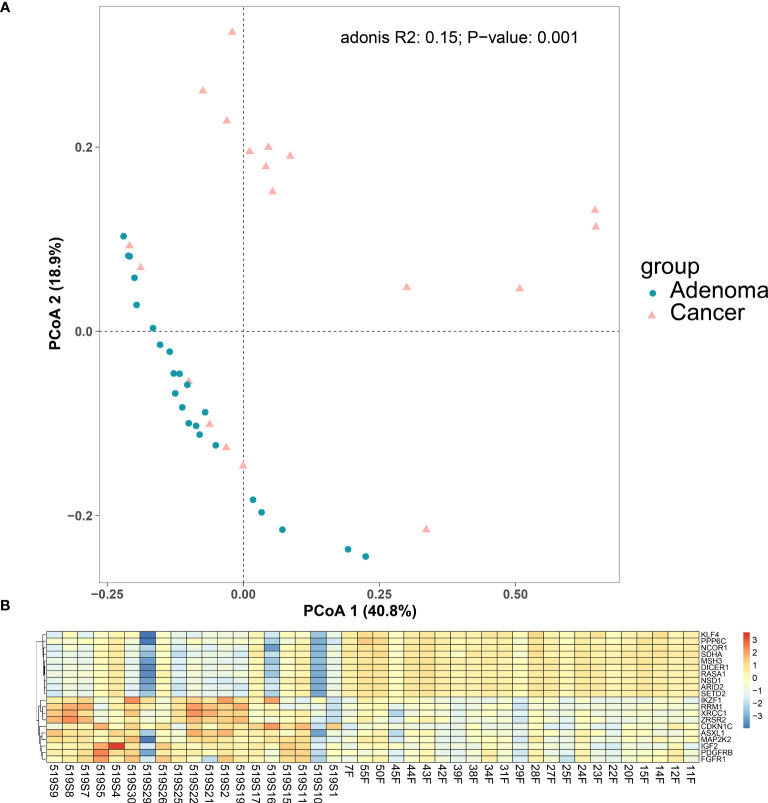
Differences between colorectal cancer group and adenoma group. **(A)** PCoA plot for the cancer group and the adenoma group. **(B)** Differentially expressed gene between the colorectal cancer group and the colorectal adenoma group. Top 10 differentially expressed genes in colorectal cancer samples (1–19 on the horizontal axis) compared with adenoma samples (20–42 on the horizontal axis).

We also plotted the PCoA for our cancer and adenoma cohorts. The PCoA showed the cancer samples and the adenoma samples could be distinguished, shown in **Figure 4A** (*p* = 0.001 using Adonis analysis). Differentially expressed gene (DEG) analysis was then applied to compare the expression profiles of colorectal adenoma and colorectal cancer, and DEGs are listed in **Supplementary Table 5** (56). Compared with the RNA expression level of the adenoma samples, 151 genes with significantly different expressions were found in the cancer samples, with 99 (65.56%) upregulated genes and 52 (34.44%) downregulated genes. The top 10 downregulated and upregulated genes are shown in **Figure 4B**. The top downregulated gene, the Kruppel-like factor 4 (*KLF4*) gene, encodes a transcription factor that belongs to the Kruppel family. It is involved in the differentiation process of epithelial cells and is thought to control the G1-to-S transition of the cell cycle following DNA damage by mediating the tumor suppressor gene p53. It is also associated with secretory meningioma and epilepsy. *KLF4* participates in signaling pathways regulating the pluripotency of stem cells and chemical carcinogenesis - receptor activation. Another downregulated gene, *MSH3*, takes part in pathways of platinum drug resistance, mismatch repair, and colorectal cancer, and it is related to colorectal cancer, endometrial cancer, and familial adenomatous polyposis. *FGFR1*, one of the upregulated genes, encodes a protein that belongs to the family of the fibroblast growth factor receptor, which is a key factor in many cancer-related pathways, such as the MAPK signaling pathway, Ras signaling pathway, and PI3K-Akt signaling pathway.

An independent RNA-seq dataset, GSE164541 containing 5 colorectal cancer samples and 5 colorectal adenoma samples was collected to confirm our DEGs. The same DEG analysis were conducted as in our dataset. 849 DEGs were found in GSE164541 and supplied in **Supplementary Table 6**. 18 common DEGs were found. We performed a hypergeometric test (see Materials and Methods for details) to assess the significance of the overlap between the DEGs identified in two datasets. Our analysis showed that the probability of observing such an overlap by chance alone was 0.0004, indicating a significant enrichment of common DEGs between the two datasets.

### 2.7 Adenoma and cancer samples could be classified using mutation profile or expression profile

**Figure 5:**
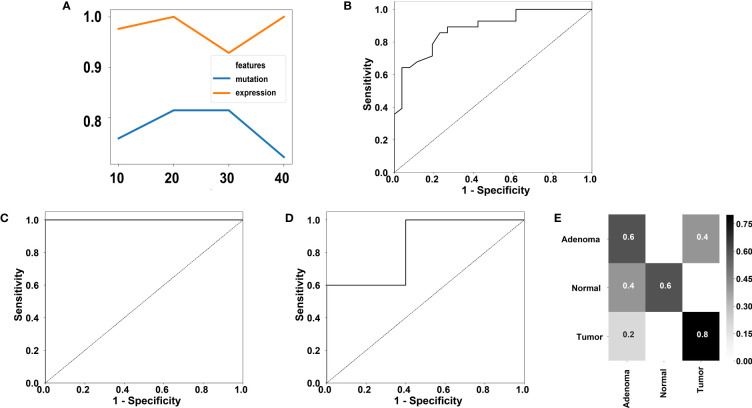
Performance of classification of colorectal adenoma and cancer using our 519-gene panel. **(A)** Accuracies using different gene number for mutation or expression profiles. **(B)** ROC using 20 genes for mutation profiles. The AUC is 0.88. **(C)** ROC using 20 genes for expression profiles. The AUC is 1.00. **(D)** ROC of the GSE16454 dataset containing 5 adenoma samples and 5 cancer samples. The AUC is 0.84. **(E)** Confusion matrix of a two-fold cross validation using the GSE164541 dataset utilizing the 20 informative genes discovered in our expression profiles.

Because some genes were differentially mutated or expressed between cancer samples and adenoma samples in this study, and this observation has also been previously reported (57), this study further investigated whether cancer samples and adenoma samples could be distinguished using the currently available molecular markers. The mutation profiles covering 411 genes from 28 cancer samples and 26 adenoma samples were used to evaluate the performance of classification for mutation. The expression profiles covering the entire 519-gene panel from 19 cancer samples and 23 adenoma samples were used to evaluate the performance of classification for expression. Tenfold cross-validation was used for both datasets. Random forest (58), a classic classification method, was used to classify the cancer samples and adenoma samples. To prevent overfitting, we utilized the random forest algorithm for feature selection and set the maximum number of selected genes equal to the sample size with a step size of 10. Thus, we used 10, 20, 30 and 40 as the candidate gene numbers for both mutation profiles and expression profiles. The performance of the different gene numbers was evaluated using a random forest classifier. The accuracies using different number of genes for the mutation profiles and the expression profiles. were shown in **Figure 5A**.

For achieving the best accuracy for the mutation profiles, 20 is the optimal gene number. The receiver operating characteristic (ROC) using 20 genes for mutation profiles is displayed in **Figure 5B**. A high accuracy of 81.48% was achieved by the 20-gene set, which contained TP53, SOX9, CCDC6, ETV6, CLTC, AGK, EML4, CUL1, BRAF, FIP1L1, PAPSS1, TTN, EGFR, ETV5, BCL2, TPM3, GATA3, SPTA1, MUC16, and ARID1A. In the cross-validation of the 20-gene set, six adenoma samples were misclassified as cancer samples, and four cancer samples were misclassified as adenoma samples.

To achieve the best accuracy for the expression profiles, 20 is the optimal gene number. The ROC using 20 genes for expression profiles is displayed in **Figure 5C**. A high accuracy of 100% was achieved by the 20-gene set, which contained PPP6C, RASA1, MSH3, NSD1, BRD4, RYBP, NCOR1, SDHA, ARID2, EP300, ASXL2, REL, FOXP1, SETD2, MAP2K4, SDHC, DICER1, IGF2, SMAD2, and ASXL1. Note that only FOXP1 is not one of the DEGs.

We further validated the 20 gene set for expression profiles in an independent dataset. We trained a model using random forest based on the 20 genes from adenoma and cancer samples and tested the model performance on the independent RNA-seq dataset, GSE164541, which contains 5 colorectal adenoma samples and 5 colorectal cancer samples. The area under curve (AUC) of classifying the independent dataset reached 0.84 (**Figure 5D**), showing the robustness of the 20 genes in classifying colorectal adenoma and cancer samples.

Additionally, the GSE164541 dataset supplied another 5 normal samples. To investigate whether the 20 genes could help to distinguish the normal samples, we performed a twofold cross-validation for the GSE164541 dataset with only the 20 informative genes found in this study’s dataset. An accuracy of 66.7% was achieved on the GSE164541 dataset and the confusion matrix was displayed in **Figure 5E**, showing the 20 genes have the potential in distinguish the normal samples.

We demonstrated that the genes contained in the 519-gene panel were sufficient for determining sample malignancy. Thus, the 519-gene panel, which could capture both DNA and RNA sequences, might help to solve the problem of early screening and monitoring while also lowering costs.

### 2.8 Correlations between expression and mutation were discovered in adenoma samples

We further investigated whether there were correlations between mutations and expressions. Three pairs were found to be strongly correlated: mutation of GATA3 with expression of IDH1 (coefficient = 0.71, *p*-adj = 0.01), mutation of EML4 with expression of ROS1 (coefficient = -0.68, *p*-adj = 0.02) and mutation of EML4 and the expression of GATA1 (coefficient = -0.66, *p*-adj = 0.03). GATA3 encodes a protein that is important regulator to T-cell development and related to cancer (59, 60). EML4 is a gene that frequently involved in fusion events (61, 62). Based on our parallel capture technique, those findings might supply us new insight for the prevention and curation of colorectal cancer.

## 3 Materials and methods

### 3.1 Case collection

Individuals were diagnosed with colorectal cancer and did not receive any treatment before sample collection. Patients with hereditary nonpolyposis colorectal cancer and a history of colorectal cancer were excluded. Based on inclusion criteria in addition to similar sex and age, samples were collected from the Shanghai Changhai Hospital, Peking Union Medical College Hospital, Jiangmen Central Hospital, and Maoming People’s Hospital along with patients’ clinical information. For this study, all patients provided informed consent.

An additional RNA-seq expression dataset, GSE164541, was collected from the Gene Expression Omnibus database (GEO) to supply adjacent normal tissue, adenoma tissue, and primary colorectal cancer tissue for each of the five patients, forming a 15-sample dataset.

To obtain the expression profile from TCGA, read counts of colorectal samples were downloaded from https://dcc.icgc.org/releases/release_26.

### 3.2 DNA/RNA extraction

DNA and RNA were extracted from the obtained case samples. DNA was extracted from both tumor/adenoma and blood. RNA was only extracted from tumor/adenoma. The Quick DNA/RNA FFPE kit (ZYMO) was used for extraction from formalin-fixed paraffin-embedded (FFPE) sections. DNA quantification was conducted by Qubit dsDNA HS Analysis Kit (LIFE) and agarose gel electrophoresis, while RNA was quantified by Qubit RNA HS Assay Kit (LIFE) and RNA 6000 Pico Kit (Agilent). The construction of DNA and RNA libraries was accomplished using the ABclonal Rapid DNA Lib prep kit and KAPA Stranded RNA-Seq Kit with RiboErase HMR, according to the manufacturer’s protocol. Covaris s220 was used to physically cut 50–200-ng DNA, followed by A-tailing, adaptor ligation, and polymerase chain reaction amplification. The total amount of DNA and RNA libraries was 500 ng, and the mixing ratio was 10:1. The mixture was hybridized at 65°C for 16–18 h using hybridization probes from Boke, followed by the use of M270 streptavidin beads for 45 min for capture. Then 15 post-capture amplification cycles were carried out to obtain the captured library.

### 3.3 Next-generation sequencing and quality control

All samples were sequenced using a panel-based NGS system that contained 519 cancer-related genes and covered 1.8 Mb of the human genome (GRCh37/hg19) (see **Supplementary Table 7** for details). A special capture technology called DNA&RNA Parallel Capture (P-Cap, patent authorization publication number: CN 110079594 B), which can obtain DNA and RNA sequences through a single capture operation, was used in the experiment. The final libraries were pooled and sequenced using the MGI-2000 sequencing platform with the paired-end 100-cycle kit. To ensure quality control, a sequencing depth of 100x was set for both DNA and RNA samples.

### 3.4 Somatic mutation calling

Mutations shown in matched blood samples were taken as germline mutations. The Microraptor (https://github.com/umich-brcf-bioinf-projects/microraptor) pipeline, which is based on GATK, was used to call somatic mutations. Trimmomatic was used to filter low-quality reads, and BWA mem was used for the mapping of reads. GATK SortSam, MarkDuplicates, CollectHsMetrics, BaseRecalibrator, ApplyBQSR, AnalyzeCovariates, CollectSequencingArtifactMetrics, Mutect2, GetPileupSummaries, CalculateContamination, FilterMutectCalls, and FilterByOrientationBias were used for mutation calling and filtrations.

### 3.5 Mutation analysis

Maftools (63) was used for multiple analysis in mutation profiles. For visualization of the mutations in samples, the oncoplot function was used. To discover the transitions/transversions relationships, the titv function was used. To determine the co-occurrence and mutually exclusive patterns in the mutations between genes, the somaticInteractions function was used, where Pair-wise Fisher’s Exact test were performed and Bonferroni correction was used to adjust the *p*-values. Based on the OncodriveCLUST algorithm, a function called oncodrive was used to detect the driver genes. Bonferroni correction was then used to adjust the *p*-values. The driver genes with FDR < 0.01 were displayed in this study.

### 3.6 Fusion detection

SEGF software (64) was used to detect the fusions in both DNA and RNA fastqs. The default parameters were used.

### 3.7 RNA expression profile calling

Mapsplice v12_07 was used to map the RNA reads onto the hg19 genome using default parameters. RSEM (v1.1.13) was used for quantification of gene and isoform abundance estimation according to TCGA GAF 2.1 files using default parameters.

### 3.8 Batch correction

To eliminate the batch effect, we used the combat function from sva package (65) for batch correction. Null model was used, which assumes equal variance across our samples and TCGA samples. After batch correction, negative values were replaced to zero.

### 3.9 Principal coordinate analysis and Adonis analysis

The dissimilarities between samples was first calculated using Bray-Curtis dissimilarity distance measure using vegdist function from vegan package (66). cmdscale function was then used to keep the first three principal coordinates. Bray-Curtis dissimilarity distance measure was chosen for the Adonis analysis using adonis2 function from vegan package.

### 3.10 Differentially expressed gene analysis

DEG analysis was performed using R package DESeq2 (56). Wald significance tests was used in DESeq function. Benjamini-Hochberg were used for controlling false discovery rate. The thresholds were set to log2 |fold change| > 1 and adjusted p-value < 0.01 for further analysis.

### 3.11 Hypergeometric test for assessing the significance of the overlap between the DEGs identified in two datasets

We employed the R function phyper to determine the significance of overlap between two sets of differentially expressed genes (DEGs). In our study, the parameters q=17, m=151, n=20827, and k=849 were used to define the size of each set and the level of overlap.

### 3.12 Classification for colorectal cancer and adenoma

Before classification, the expression data, the read counts were normalized for each sample. For mutation data, the mutation counts on each gene were used as features.

For gene selection, the scikit-learn package (67) was used to implement a 100-estimator random forest to get the importance scores of each gene. and the Gini impurity was used to measure the quality of a split. The feature number used by each estimator is square root of total feature.

For classification, we implement a 100-estimator random forest classification model with the same hyperparameters. The reason we chose random forest as the classification algorithm is that (a) each tree of random forest process a random subset of features and samples, alleviating the problem of overfitting; (b) the ensemble of trees could alleviate the curse of dimensionality.

### 3.13 Correlation calculation between mutations and expressions

The gene mutation profile was processed by indicator function, which will output whether the gene has mutation (output = 1) or not (output = 0). To decrease false discoveries, genes that mutated in less than 5 samples were excluded. Pearson correlation from scipy package (68) was used to calculate the correlation between the mutation of one gene and the expression of another gene. Benjamini & Hochberg method from statsmodels package (69) was used for false discovery.

## 4 Discussion

In this study, 54 FFPE samples were collected from patients with colorectal adenoma or colorectal cancer as well as the matched blood samples to explore the molecular characteristics of colorectal adenomas and the relationship to colorectal cancer. P-Cap was employed to capture DNA and RNA simultaneously, and the NGS results were analyzed. Mutations of the *APC*, *TTN*, *MUC16*, and *KRAS* genes were high in frequency in both colorectal adenoma and colorectal cancer samples. However, some differences were identified, such as the relatively high occurrences of mutations in *SOX9* and *ARID5B* in the colorectal adenoma samples, and the relatively high mutation frequencies of *TP53* and *SPTA1* in the colorectal cancer samples.

The underlying driver genes of colorectal adenoma and colorectal cancer were identified by mining DNA sequencing data. *GNAQ* and *KRAS* were identified as driver genes in both colorectal adenoma and colorectal cancer samples. *GNAQ* was a potential driver in colorectal cancer. The mutated *GNAQ* gene is a proto-oncogene of uveal melanoma, and the activation of the pathway containing the mutated *GNAQ* may be the main cause of uveal melanoma (70, 71). *KRAS* is an important “switch” in intracellular signaling and is most closely related to the occurrence and development of tumors; it is also a drug target. The activate mutation of *KRAS* is one driving factor in metastatic progression and has been reported in a number of studies. The mutated *KRAS* can drive the invasion and maintenance of metastasis of colorectal cancer, and it may also be a potential biomarker and therapeutic target for metastatic colorectal cancer (72, 73).

Our finding of the additional fusion detected only in RNA data highlights the complementary roles of DNA and RNA in the detection of fusion events. Unlike DNA, RNA can reveal information on splicing patterns and post-transcriptional modifications that can affect gene expression and alter the protein structure and function. Thus, we could detect additional fusion in RNA (74). Moreover, as previously reported by Hechtman et al., DNA plays a crucial role in detecting fusions, which could complement RNA-based detection methods (75). Therefore, the combination of DNA and RNA sequencing can offer a more comprehensive and accurate characterization of fusion events in cancer, which can promote the development of targeted therapies and improve the prognosis for patients.

Since there were differences in both mutation profiles and expression profiles between the adenoma group and the cancer group, a classification model was designed to distinguish between colorectal adenoma samples and colorectal cancer samples, and it performed well. It is worth noting that the model trained on the expression profile performed better than the model trained on the mutation profile. RNA seems to be more informative regarding sources of tissue, which has been addressed by other studies (3, 19, 20, 76, 77). In the future, the sample amount should be enlarged to verify the classification model proposed in this study. Although some previous studies also used random forest in small datasets that contained less than 100 samples (78, 79), our classification model might not be robust since our study’s dataset does not meet the requirement of event per variable. In addition, more efforts should be made to simplify the panel for the expression profile to detect colorectal cancer more inexpensively. Lin et al. developed a 20-gene panel using only mutation profiles for classifying adenoma and cancer at an accuracy of 85.46%, which is 3.98% higher than the accuracy of our proposed model (81.48%) (23). The feature used in their work was the severe consequences for each gene, whereas we used a simpler feature, the mutation count for each gene. We also found that *TP53* and *SOX9* were both in our 20-gene mutation gene set and their 20-gene set. Furthermore, it is possible to use the interaction term between mutations, such as co-occurrence and mutually exclusive relationships. However, since the interaction term affects the number of parameters, the model must be carefully designed. Though our model performed slightly worse than the model by Lin et al. within only mutation profiles, our method could still supply high classification accuracy from expression profiles, which also provided insights from the RNA level. In the future, it might be possible to achieve better prediction by integrating more types of patients’ data, such as histopathological images and microbes, as demonstrated in colorectal and other cancers (80–84).

In this study, we investigated correlations between mutations and expressions to gain insights into colorectal cancer prevention and curation. Our results showed strong correlations between three pairs of mutations and expressions: mutation of GATA3 with the expression of IDH1, mutation of EML4 with the expression of ROS1, and mutation of EML4 with the expression of GATA1. These findings provide new insights into the molecular mechanisms that underlie colorectal cancer development and progression, especially in relation to gene-gene interactions. Moreover, our parallel capture technique offers an efficient and reliable way to investigate these correlations, which can lead to the discovery of new biomarkers and therapeutic targets. Compared to separate capture technique, our technique minimizes the differences between the tissues for DNA sequencing and RNA sequencing caused by heterogeneity (39). Taking advantage of this technique, we could generate more accurate results in analysis requiring both DNA and RNA, such as correlation analysis. Further studies are needed to validate our findings and to explore the clinical implications of these novel insights.

In conclusion, using the proposed 519-gene DNA and RNA P-Cap sequencing platform, mutation and expression profiles both revealed the molecular characteristics and correlations of these characteristics between colorectal adenoma and colorectal cancer. These molecular characteristics, especially the expression profiles, can be used as early monitoring indicators to predict the occurrence and development of colorectal adenoma to cancer. The findings of this study will be valuable for developing molecular prevention and surveillance programs for colorectal cancer. Our results are based on a cross-comparison, and we will continue to follow the prognosis of patients in the cohort to further explore the risk of cancer development in adenoma patients with similar genetic alterations to those of colorectal cancer patients. Should the cancer risk of adenoma patients with these mutations increase, further attention and monitoring of progress will be done. In the future, it will be helpful to study colorectal adenoma and colorectal cancer using single cell techniques (85–89), as these diseases are both very heterogeneous.

## Data availability statement

The datasets presented in this study can be found in online repositories. The names of the repository/repositories and accession number(s) can be found below: https://www.ncbi.nlm.nih.gov/, PRJNA783877.

## Ethics statement

This study was approved by the Ethics Committee of Peking Union Medical College Hospital (Ethical No. HS-1686), the Ethics Committee of Shanghai Changhai Hospital (Ethical No. CHEC2018-174) and the Ethics Committee of NanFang Hospital of Southern Medical University (Ethical No. NFEC-2018-157). The participants [legal guardian/next of kin] provided written informed consent to participate in this study. The patients/participants provided their written informed consent to participate in this study.

## Author contributions

GT, ZL, and YB conceived the project. PP, JL, and XL implemented the experiments. BW, CX, and YH analyzed the data. XS, HY, and LFC prepared the figures. XT, HW, and LYC prepared the data and performed literature search. PP, JL, and BW wrote the manuscript. All authors contributed to the article and approved the submitted version.
