# Supplementary figures and images for: Molecular characterization of colorectal adenoma and colorectal cancer via integrated genomic transcriptomic analysis
Source: Front Oncol. 2023 Jul 21;13:1067849. doi: 10.3389/fonc.2023.1067849 (PMC10401844; doi:10.3389/fonc.2023.1067849)

adonis R2: 0; P-value: 0.147

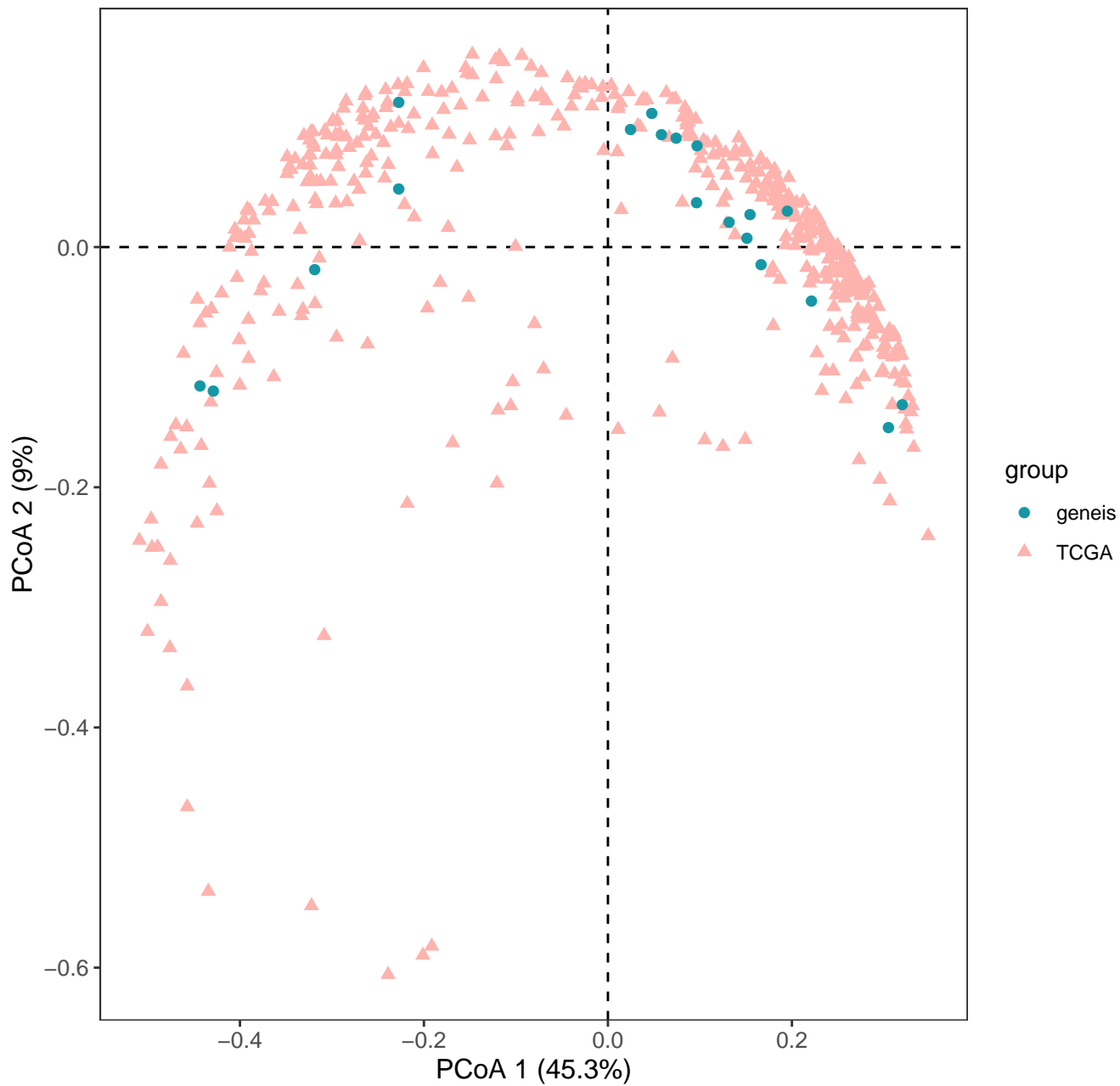

Supplement: Supplementary file 1 [file Image_1.pdf]
